# CD72, a new immune checkpoint molecule, is a novel prognostic biomarker for kidney renal clear cell carcinoma

**DOI:** 10.1186/s40001-023-01487-8

**Published:** 2023-11-18

**Authors:** Lv Tian, Yiming Wang, Zhiyuan Zhang, Xuechao Feng, Fengjun Xiao, Minru Zong

**Affiliations:** 1 Department of Rehabilitation, China-Japan Union Hospital of Jilin University, Changchun, 130033 China; 2 School of Nursing, Jilin University, Changchun, China; 3 School of Life Sciences, Northeast Normal University, Changchun, China; 4 Beijing Institute of Radiation Medicine, Beijing, 100850 China

**Keywords:** Kidney renal clear cell carcinoma, CD72, Immune checkpoint, Immune infiltration, Prognosis, Biomarker

## Abstract

**Background:**

The incidence and mortality of clear cell carcinoma of the kidney increases yearly. There are limited screening methods and advances in treating kidney renal clear cell carcinoma (KIRC). It is important to find new biomarkers to screen, diagnose and predict the prognosis of KIRC. Some studies have shown that CD72 influences the development and progression of colorectal cancer, nasopharyngeal cancer, and acute lymphoid leukemia. However, there is a lack of research on the role of CD72 in the pathogenesis of KIRC. This study aimed to determine whether CD72 is associated with the prognosis and immune infiltration of KIRC, providing an essential molecular basis for the early non-invasive diagnosis and immunotherapy of KIRC.

**Methods:**

Using TCGA, GTE, GEO, and ImmPort databases, we obtained the differentially expressed mRNA (DEmRNA) associated with the prognosis and immunity of KIRC patients. We used the Kruskal–Wallis test to identify clinicopathological parameters associated with target gene expression. We performed univariate and multivariate COX regression analyses to determine the effect of target gene expression and clinicopathological parameters on survival. We analyzed the target genes' relevant functions and signaling pathways through enrichment analysis. Finally, the correlation of target genes with tumor immune infiltration was explored by ssGSEA and Spearman correlation analysis.

**Results:**

The results revealed that patients with KIRC with higher expression of CD72 have a poorer prognosis. CD72 was associated with the Pathologic T stage, Pathologic stage, Pathologic M stage, Pathologic N stage, Histologic grade in KIRC patients, Laterality, and OS event. It was an independent predictor of the overall survival of KIRC patients. Functional enrichment analysis showed that CD72 was significantly enriched in oncogenic and immune-related pathways. According to ssGSEA and Spearman correlation analysis, CD72 expression was significantly associated with tumor immune cells and immune checkpoints.

**Conclusion:**

Our study suggests that CD72 is associated with tumor immunity and may be a biomarker relevant to the diagnosis and prognosis of KIRC patients.

## Introduction

Renal cell carcinoma (RCC) is one of the ten most common cancers; 73,750 new cases of RCC and 14,830 RCC deaths were reported in the U.S. in 2020 [1]. Kidney clear cell carcinoma (KIRC) is the main pathologic subtype of RCC [2]. Despite our great advances in diagnosis, screening, surgery, and treatment, the clinical outcomes of KIRC are still unsatisfactory [3]. The prognosis for advanced KIRC is extremely poor due to its inherent resistance to radiotherapy and chemotherapy, and the challenge for clinical management lies in treating the poor prognosis caused by radiotherapy and chemotherapy resistance [4]. KIRC has an immunogenic tumor microenvironment (TME) that contains a variety of tumor-infiltrating T lymphocytes [5]. Features of the tumor microenvironment heavily affect disease biology and may affect responses to systemic therapy [6–9]. In recent years, advances in immunotherapy, particularly immune checkpoint blockade (ICB) and engineered T cells have revolutionized cancer treatment [10]. ICB or ICB plus TKIs targeting programmed cell death 1 (PD-1), programmed cell death ligand 1 (PD-L1), and cytotoxic T-lymphocyte-associated antigen 4 (CTLA4) are now the standard of care for RCC [11, 12]. In the era of ICB, understanding immunogenic TME will help to find new therapeutic strategies in KIRC management.

CD72 is a type II membrane protein expressed mainly in B cells and is a member of the C-type lectin superfamily [13]. CD72 contains a C-type lectin-like structural domain (CTLD) and an immunoreceptor tyrosine-based inhibitory motif (ITIM) [14, 15]. CD72 overexpression can negatively regulate BCR signaling in B cell lines by recruiting phosphatase 1 (SHP-1) of the SH2 structural domain to phosphorylated ITIM [16]. CD72 functions similarly to the inhibitory co-receptor CD22 in down-regulating B-cell receptor (BCR) signaling and functioning as a molecular switch determining whether apoptosis or proliferation occurs in antigen-stimulated B cells [14]. Furthermore, CD72 and SEMA4D (CD100) interaction enhances the activation of B cells and dendritic cells (DCs) [17]. Recent studies have shown that CD72 is closely associated with developing various immune-related diseases. For example, CD72 is strongly associated with developing systemic lupus erythematosus (SLE) [18–20] in autoimmune diseases. CD72 is also closely associated with the tumor microenvironment [21]. Several studies have shown that CD72 is a marker for progenitor cell B-cell leukemia and a new marker for detecting microscopic residual disease in acute lymphoblastic leukemia [22, 23]. In addition, CD72 has been identified as a prognostic gene in the tumor microenvironment of colorectal cancer [24, 25]. CD72 may be an independent predictor of prognosis in nasopharyngeal carcinoma patients [26]. However, there are no studies on the role of CD72 in immune infiltration in KIRC. Therefore, this study aimed to investigate the relationship between CD72 and immune infiltration and prognosis of KIRC and to provide an important molecular basis for the early non-invasive diagnosis and immunotherapy of KRIC.

## Materials and methods

### Data sources and preprocessing

We used the ImmPort (https://www.immport.org/shared/home) database to obtain immune-related genes [27]. The differential RNAseq expression data of CD72 in pan-cancer were obtained from UCSC XENA (https://xenabrowser.net/datapages/) in the TPM format of the TCGA and GTEx processed uniformly by the Toil process [28]. The differential RNAseq expression data of CD72 in unpaired and paired samples were in level 3 HTSeq‐FPKM format from the TCGA (https://portal.gdc.cancer.gov/) KIRC project. FPKM (Fragments Per Kilobase per Million) format RNAseq data were converted to TPM (transcripts per million reads) format and log2 transformed. All final analyses were performed using data in TPM format. The differential analysis data for CD72 in dataset GSE40435, GSE53757 [29, 30] were downloaded from the GEO database using the GEOquery package (version 2.54.1) [31]. These data were obtained by removing probes corresponding to multiple molecules. When probes corresponding to the same molecule were encountered, only the probe with the largest signal value was retained. Then the data were normalized again by the normalize Between Arrays function of the limma package (version 3.42.2) [32]. All statistical analyses and visualizations were performed using R (version 4.2.1).

### Cell culture

HK-2 cells were cultured in a specialized medium (Pricella, CM-0109). KIRC cell line (786-O, ACHN) was cultured in high sugar DMEM (Sigma) with 10% FBS (ExCell Bio) and 1% P/S (Solarbio). Both cell lines were cultured at 37 °C in a humidified environment containing 5% CO2.

### Real-time PCR

After the cells were washed with PBS, Trizol and chloroform were added sequentially, and after repeated blowing and mixing, the cells were left to stand for 10 min and then centrifuged at 12,000 rpm for 15 min at 4° C. The supernatant was transferred to a new EP tube. An equal volume of isopropanol was added to mix it well, and after centrifugation, the RNA precipitates were cleaned with 75% ethanol and then lysed after air-drying, and the concentration was determined. The reaction system and procedures were constructed according to the instructions of the reverse transcription kit from Beijing TransGen Biotech. Real-time fluorescence quantification was performed by the SYBR Green method, and the reaction conditions were as follows: pre-denaturation at 94 ℃ for 30s, 94 ℃ for 5s, and 60 ℃ for 30s, for a total of 40 cycles. The relative expression levels of target genes were calculated using the 2-ΔΔCt method [33]. The primers were synthesized by Shanghai Sangon Biological Company: β-actin Forward primer 5′-CCTGGCACCCAGCACAAT-3′, reverse primer 5′-GGGCCGGACTCGTCATAC-3′; CD72-3 forward primer 5′-TCCGTCGGGGATGGATAATGC-3′, reverse primer 5′-TGCGTTGTGTATCATCAGTCAA-3′.

### Differential expression analysis of CD72

The expression profiles of CD72 across cancers were analyzed for differences using the Mann–Whitney *U* test (Wilcoxon rank sum test). The Shapiro–Wilk normality test was used to test the normality of the CD72 expression data in paired samples, unpaired samples, and GSE40435, GSE53757, and the independent samples *t* test was used to analyze the differences in the data in unpaired samples. The paired-samples *t* test was used to analyze the differences in the data in paired samples, and the Mann–Whitney *U* test (Wilcoxon rank sum test) was used to analyze the data variance in GSE40435, GSE53757. The results of all the above analyses were visualized using ggplot2 (version 3.3.3) and were considered statistically significant when *P* < 0.05.

### Differential analysis of CD72 protein expression levels in KIRC

Immunohistochemical staining images of CD72 in KIRC and normal tissue sections were downloaded using the HPA database (https://www.proteinatlas.org/).

### Analysis of DEmRNA

We used the DESeq2 package [version 1.36.0] to perform gene differential analysis and CD72 single gene correlation analysis of KIRC in the TCGA database [34]. The results of the genetic difference analysis were used to generate volcano plots using ggplot2 software [version 3.3.3] [35]. |(LogFC)|> 1 and *p*.adj < 0.05 were used as thresholds for differentially expressed genes (DEGs). CD72 single gene co-expression heatmaps were generated by the ggplot2 [version 3.3.6] package using genes in positively and negatively correlated top15.

### Identification of target genes associated with immunity

We used the ImmPort (https://www.immport.org/shared/home) database to obtain immune-associated genes. Prognosis-related DEmRNA and immune-related genes were then derived using VennDiagram [version 1.7.3] overlap analysis. Finally, we identified CD72 as the target gene.

### Clinical correlation analysis, survival prognosis analysis

The survival data of KIRC patients were statistically analyzed using the survival package (version 3.2‐10), and the results were visualized using the survival package (version 0.4.9) to plot KIRC patients' overall survival (OS), disease‐specific survival (DSS), and progression‐free interval (PFI) of Kaplan–Meier survival curves. We then performed a subgroup analysis of Kaplan–Meier survival curves in KIRC patients, analyzing clinicopathological factors such as age, gender, Pathologic T stage, serum calcium, and hemoglobin. We used these clinicopathological factors to calculate their correlation with CD72 expression and visualized the calculations using ggplot2 (version 3.3.3). ROC analysis of the data was performed with the pROC software package (version 1.17.0.1) to determine the accuracy of CD72 in predicting prognosis. Finally, logistic analysis of different clinicopathologic factors and CD72 expression were performed using the Survival Package (version 3.2-10). All prognostic data for the above survival analyses were obtained from a paper in cell [36].

### Functional enrichment analysis

The single gene differential analysis results were analyzed for GO, KEGG, and GSEA functional enrichment using the clusterProfiler software package (version 3.14.3) [37]. Gene ID conversion was performed using the org.Hs.eg.db package (version 3.10.0), and the *Z* score was calculated using the GOplot package (version 1.0.2) [38], which scores the relevance of CD72 to the enrichment pathway. The reference gene set used for GSEA is c2.cp.all.v2022.1.Hs.symbols.gmt (All Canonical Pathways) [39], and the results were significantly enriched if they met the conditions of false discovery rate (FDR) < 0.25 and *p*.adjust < 0.05. All the above analyses were visualized using ggplot2 (version 3.3.3).

### Immunocorrelation analysis of CD72

Relative infiltration levels of 24 immune cells were analyzed using the GSVA software package (version 24.1.34) [40]. For the immune infiltration algorithm, ssGSEA was employed, and Spearman correlation analysis was applied. Markers for 24 immune cells were obtained from an immunity study [41]. The samples were then divided into low and high-expression groups based on CD72 expression, and enrichment scores for various immune cell infiltrates in different subgroups were calculated and analyzed using the GSVA software package (version 1.34.0). Correlations between CD72 and immune cells and the expression of immune checkpoint-programmed cell death protein 1 (PD-1)-PDCD1, cytotoxic T-lymphocyte-associated protein 4 (CTLA4), and programmed cell death ligand 1 (PD-L1) were assessed using Spearman analysis. Finally, the correlation between immune cell infiltration and CD72 expression was visualized by analyzing immune cells with statistically significant relative infiltration (*P* < 0.05). The analysis results were visualized with the ggplot2 package (version 3.3.3) [42].

### Statistical analysis

Data are expressed as mean ± standard deviation (mean ± SD). Student's t test analyzed differences in CD72 expression in KIRC tumor tissues and adjacent tissues. One-way analysis of variance (ANOVA) was used for comparison between multiple groups. The Mann–Whitney *U* test was used to analyze the correlation between CD72 expression and clinical data of KIRC patients. Statistical plots were completed using GraphPad Prism 8. *P* < 0.05 was considered statistically significant.

## Results

### Acquisition of immune-related DEmRNAs

**Fig. 1:**
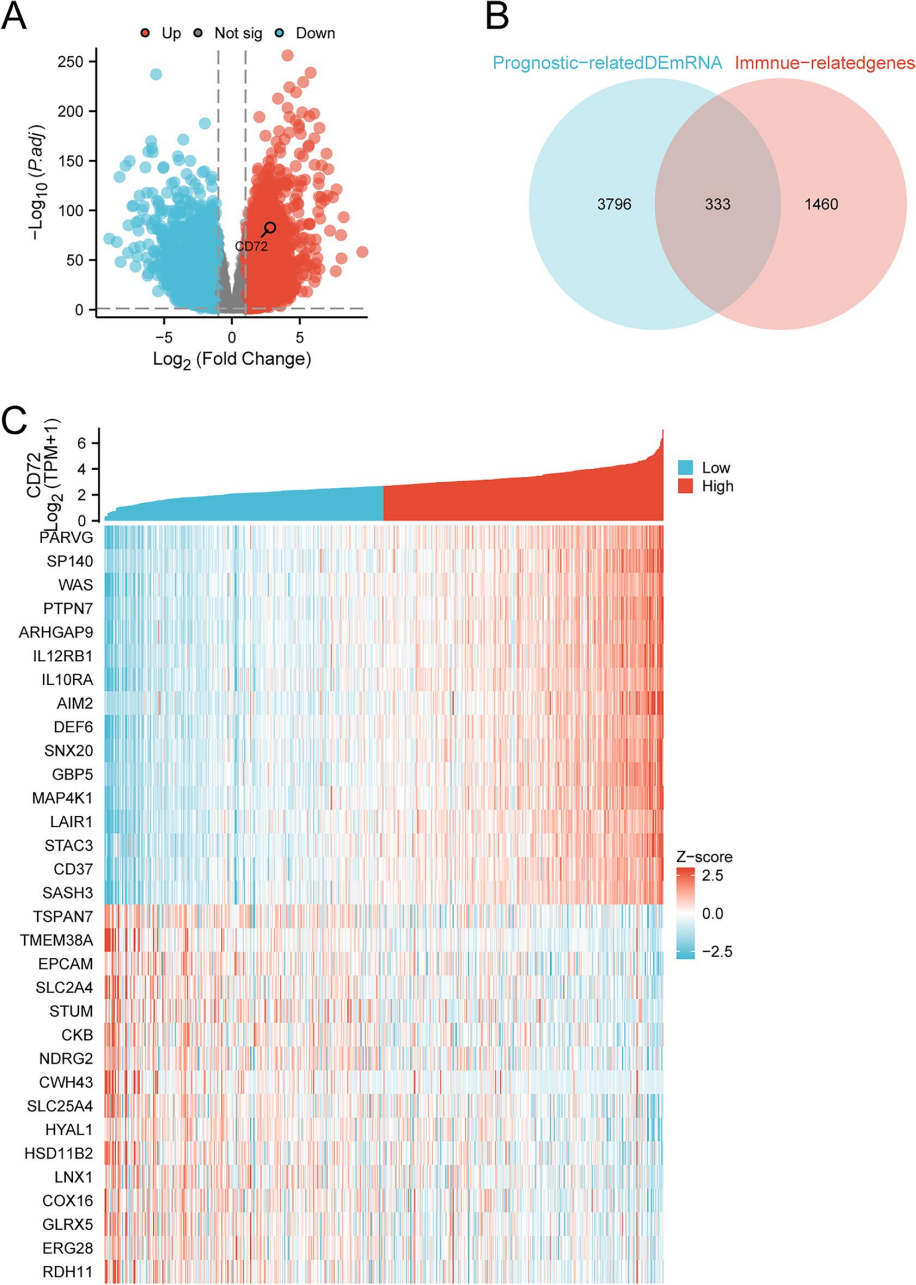
Distributions of mRNA in KIRC and identification of DEmRNA association with immunity and prognostic of KIRC patients

We found 19,596 DEmRNAs between 541 KIRC and 72 kidney normal tissues. The volcano distribution is shown in Fig. 1A. We downloaded immune-related genes from the ImmPort database. We used Venn overlap analysis to get the overlapping target genes between immune-related genes and DEmRNAs associated with the prognosis of KIRC patients. The results showed 333 overlapping target genes, such as CD72, TNFAIP3, CETP, IL18R1, HTR3A, DEFB124, and RBP7 (Fig. 1B). Through comprehensive comparison, we finally chose CD72 as the target gene. Figure 1C shows a heat map of CD72 and its co-expressed mRNAs. The first 15 genes had a positive correlation with the expression of CD72, and the last 15 genes had a negative correlation with CD72.

### A strong correlation exists between high CD72 expression and poor prognosis in KIRC

**Fig. 2:**
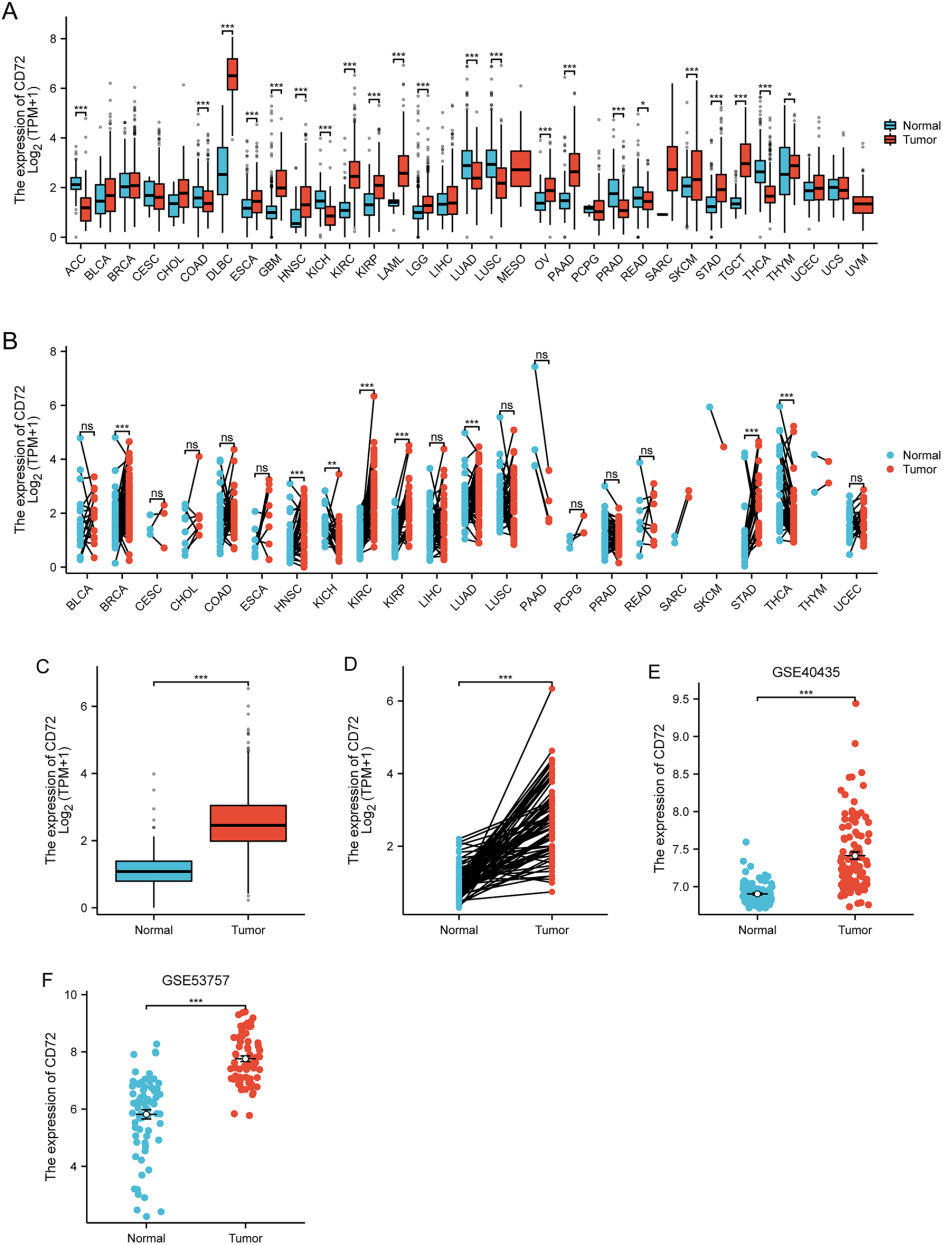
Differential expression of CD72 in pan-cancer and KIRC

Figure 2A and B shows the expression of CD72 in the unpaired sample database UCSC XENA versus the paired database TCGA pan-cancer, respectively. The CD72 expression level of KIRC was higher than in kidney tissues (Fig. 2C, D). We then validated the results in the TCGA database using the datasets GSE40435, GSE53757 from the GEO database, and the results are shown in Fig. 2E, F.

### Correlation of CD72 expression with clinicopathologic parameters

**Fig. 3:**
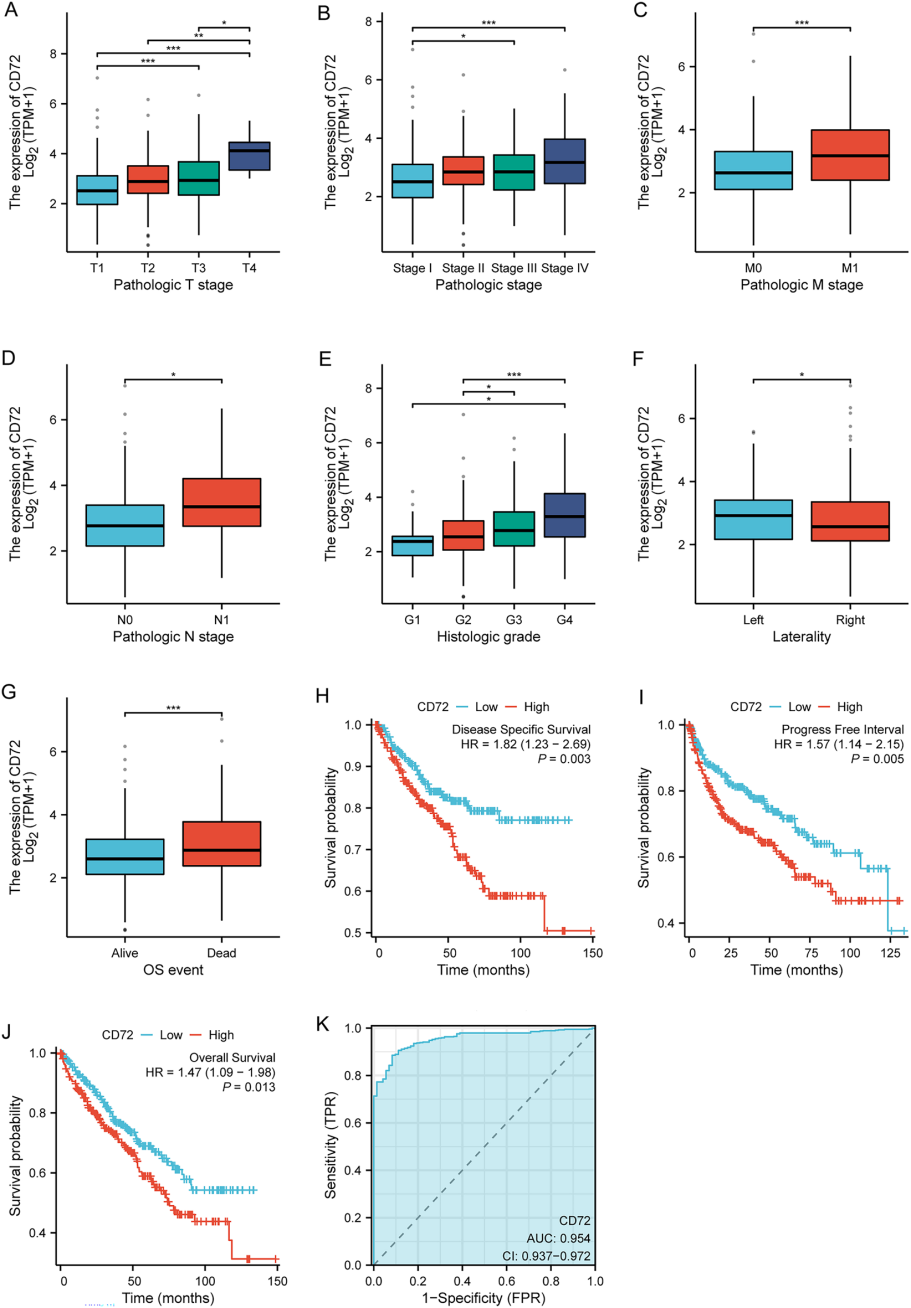
Clinical correlation analysis of CD72 expression

We analyzed the relationship between CD72 expression and various clinical characteristics of KIRC patients. Chisq test and Yates' correction were used to correlate clinicopathologic factors and CD72 expression, as shown in Table 1. Chisq test showed that CD72 was associated with Pathologic T stage (*P* < 0.001), Pathologic M stage (*P* < 0.001), Pathologic stage (*P* < 0.001), Race (*P* = 0.024), Histologic grade (< 0.001), Laterality (*P* < 0.001) were associated. The results of the logistic analysis are shown in Table 2. As shown in Fig. 3A–G, according to the Kruskal–Wallis Test and Dunn's test, CD72 expression correlated with KIRC patients' Pathologic T stage, Pathologic stage, Pathologic M stage, Pathologic N stage, Histologic grade, Laterality, and OS event were correlated (*P* < 0.05). According to survival analysis, as shown in Fig. 3H–J, high expression of CD72 was associated with poorer overall survival (OS), disease-specific survival (DSS), and progression-free interval (PFI) in KIRC patients. An AUC of 0.954 shows that KIRC may serve as a potential diagnostic biomarker (Fig. 3K).

**Table 1:** CD72 expression associated with clinicopathological characteristics (baseline data sheet)

Characteristics	Low expression of CD72	High expression of CD72	*P* value
*n*	270	271	
Pathologic T stage, *n* (%)			< 0.001
T1	164 (30.3%)	115 (21.3%)	
T2	30 (5.5%)	41 (7.6%)	
T3	76 (14%)	104 (19.2%)	
T4	0 (0%)	11 (2%)	
Pathologic N stage, *n* (%)			0.091
N0	113 (43.8%)	129 (50%)	
N1	4 (1.6%)	12 (4.7%)	
Pathologic M stage, *n* (%)			< 0.001
M0	227 (44.7%)	202 (39.8%)	
M1	25 (4.9%)	54 (10.6%)	
Pathologic stage, *n* (%)			< 0.001
Stage I	161 (29.9%)	112 (20.8%)	
Stage II	26 (4.8%)	33 (6.1%)	
Stage III	57 (10.6%)	66 (12.3%)	
Stage IV	25 (4.6%)	58 (10.8%)	
Primary therapy outcome, *n* (%)			0.163
PD	4 (2.7%)	7 (4.8%)	
SD	5 (3.4%)	1 (0.7%)	
PR	2 (1.4%)	0 (0%)	
CR	71 (48.3%)	57 (38.8%)	
Gender, *n* (%)			0.054
Female	104 (19.2%)	83 (15.3%)	
Male	166 (30.7%)	188 (34.8%)	
Race, *n* (%)			0.024
Asian	1 (0.2%)	7 (1.3%)	
Black or African American	35 (6.6%)	22 (4.1%)	
White	233 (43.6%)	236 (44.2%)	
Age, *n* (%)			0.764
< = 60	136 (25.1%)	133 (24.6%)	
> 60	134 (24.8%)	138 (25.5%)	
Histologic grade, *n* (%)			< 0.001
G1	11 (2.1%)	3 (0.6%)	
G2	136 (25.5%)	100 (18.8%)	
G3	92 (17.3%)	115 (21.6%)	
G4	24 (4.5%)	52 (9.8%)	
Serum calcium, *n* (%)			0.366
Low	114 (31.1%)	90 (24.5%)	
Normal	74 (20.2%)	79 (21.5%)	
Elevated	5 (1.4%)	5 (1.4%)	
Hemoglobin, *n* (%)			0.093
Low	124 (26.9%)	140 (30.4%)	
Normal	106 (23%)	86 (18.7%)	
Elevated	4 (0.9%)	1 (0.2%)	
Laterality, *n* (%)			< 0.001
Left	105 (19.4%)	148 (27.4%)	
Right	164 (30.4%)	123 (22.8%)

**Table 2:** The logistic analysis of clinicopathological parameters in patients with KIRC

Characteristics	Total (*N*)	OR (95% CI)	*P* value
Pathologic T stage (T1 vs. T2 & T3 & T4)	541	0.476 (0.338–0.671)	**< 0.001**
Pathologic N stage (N1 vs. N0)	258	2.628 (0.8248.378)	0.102
Pathologic M stage (M1 vs. M0)	508	2.427 (1.4574.045)	**< 0.001**
Primary therapy outcome (CR vs. PD & SD & PR)	147	1.104 (0.4162.927)	0.843
Gender (male vs. female)	541	1.419 (0.9942.026)	0.054
Laterality (right vs. left)	540	0.532 (0.3780.749)	**< 0.001**
Pathologic stage (Stage I & Stage II vs. Stage III & Stage IV)	538	0.513 (0.3600.730)	**< 0.001**
Race (White vs. Asian & Black or African American)	534	1.257 (0.7462.118)	0.389
Age (< = 60 vs. > 60)	541	0.950 (0.6781.330)	0.764
Histologic grade (G1 & G2 vs. G3 & G4)	533	0.487 (0.3440.688)	**< 0.001**
Serum calcium (Low vs. Normal & Elevated)	367	0.742 (0.4911.122)	0.158
Hemoglobin (Low vs. Normal & Elevated)	461	1.428 (0.9852.068)	0.060

### Subgroup analysis of survival prognosis of UNC93B1 expression

**Fig. 4:**
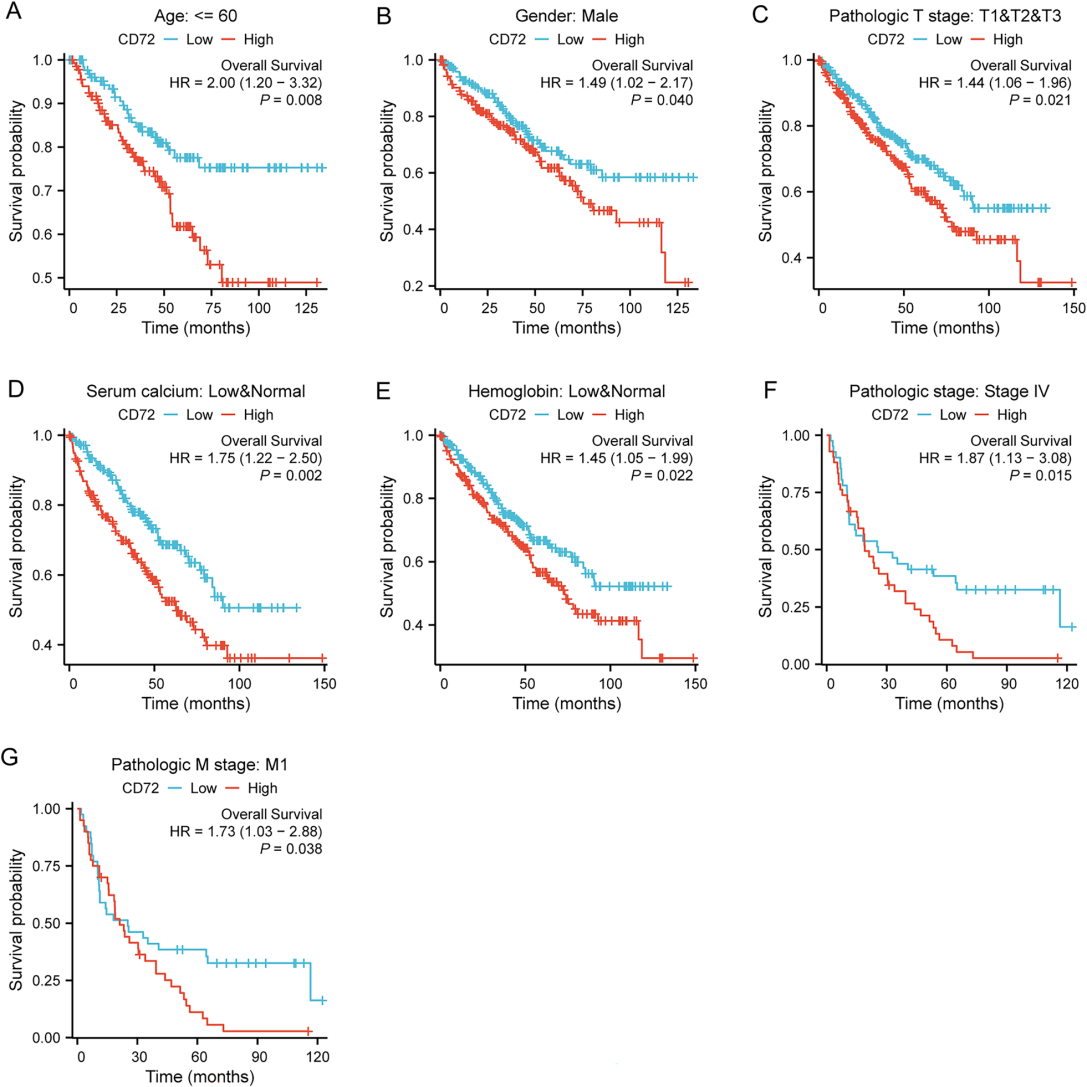
Subgroup prognostic analysis of survival and CD72 expression

Figure 4A–I shows the survival of patients with high or low CD72 expression in different KIRC subgroups.

The results showed that the subgroup of age not older than 60 (HR = 2.00 (1.20–3.32), *P* = 0.08), the subgroup of sex male (HR = 1.49 (1.02–2.17), *P* = 0.040, the subgroup of T1 & T2 & T3 of T stage (HR = 1.44 (1.06–1.96), *P* = 0.021), Low & Normal subgroup of Serum calcium (HR = 1.75 (1.22–2.50), *P* = 0.040), Low & Normal subgroup of Hemoglobin: (HR = 1.45 (1.05–1.99), *P* = 0.022), Stage IV subgroup of Pathologic stage (HR = 1.87 (1.13–3.08), *P* = 0.015), M1 subgroup of M stage (HR = 1.73 (1.03–2.88), *P* = 0.038) were associated with increased CD72 expression and poor overall survival.

### Functional enrichment analysis of CD72 in KIRC

**Fig. 5:**
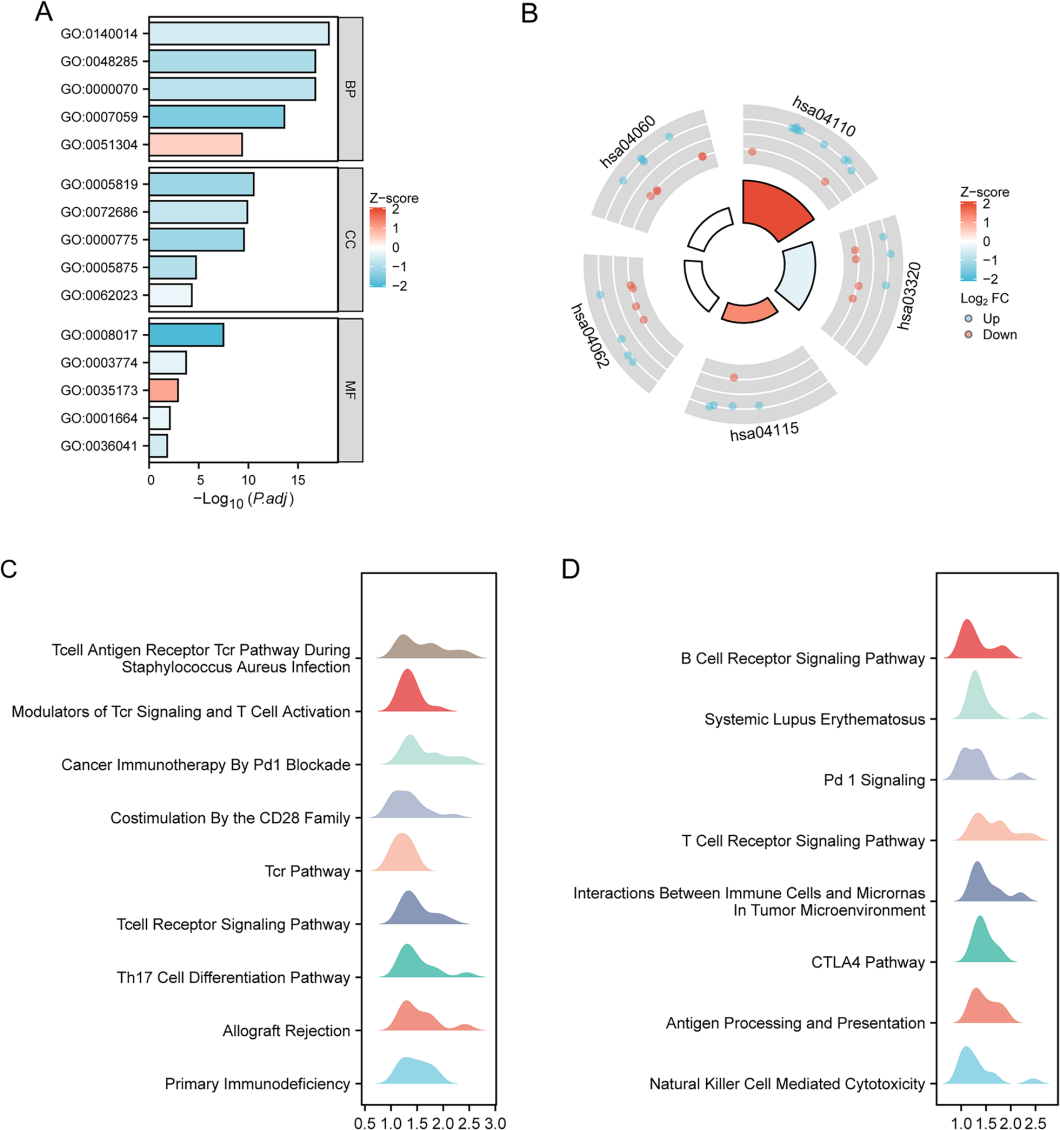
Functional enrichment analysis of CD72 in KIRC

GO, KEGG, and GSEA enrichment analyses were performed using the single gene differential analysis results shown in Fig. 5. GO analysis showed that CD72 was functionally associated with microtubule binding, cytoskeletal motor activity, histone kinase activity, G protein-coupled receptor binding, and long-chain fatty acid binding (Fig. 5A and Table 3). Figure 5B and Table 4 show the results of KEGG analysis that CD72 is associated with signaling pathways such as the Cell cycle, PPAR signaling pathway, p53 signaling pathway, Chemokine signaling pathway, and Cytokine–cytokine receptor interaction. Z scores reflect, to some extent, the relevance of CD72 to these pathways. Negative Z scores indicate a negative correlation, and positive Z scores indicate a positive correlation. Figure 5C, D shows the enrichment and grading results of GSEA, indicating that CD72 is closely associated with signaling pathways such as the Pd 1 signaling pathway, the CTLA4 pathway, the Th17 cell differentiation pathway, the B-cell receptor signaling pathway, costimulation of the CD28 family, primary immune deficiencies, cancer immunotherapies via Pd1 blockade, regulators of TCR signaling, and T-cell activation, as well as other genes associated with tumorigenesis, invasion, and angiogenesis.

**Table 3:** GO analysis

Ontology	ID	Description	Gene ratio	Bg ratio	*P *value	*p*.adjust	*Z* score
BP	GO:0140014	Mitotic nuclear division	31/197	293/18800	2.61e−22	7.82e−19	−0.5388159
BP	GO:0000070	Mitotic sister chromatid segregation	24/197	171/18800	2.26e−20	1.83e−17	− 0.8164966
BP	GO:0048285	Organelle fission	36/197	493/18800	2.44e−20	1.83e−17	− 1.0000000
BP	GO:0007059	Chromosome segregation	28/197	348/18800	4.75e−17	2.37e−14	− 1.5118579
BP	GO:0051304	Chromosome separation	14/197	97/18800	1.58e−12	4.3e−10	0.5345225
CC	GO:0005819	Spindle	26/203	402/19594	9.72e−14	2.88e−11	− 1.1766968
CC	GO:0072686	Mitotic spindle	17/203	160/19594	8.73e−13	1.29e−10	− 0.7276069
CC	GO:0000775	Chromosome, centromeric region	19/203	227/19594	2.81e−12	2.77e−10	− 1.1470787
CC	GO:0005875	Microtubule-associated complex	11/203	160/19594	9.09e−07	1.92e−05	− 0.9045340
CC	GO:0062023	Collagen-containing extracellular matrix	17/203	429/19594	2.56e−06	5.05e−05	− 0.2425356
MF	GO:0008017	Microtubule binding	19/192	272/18410	7.14e−11	3.28e−08	− 2.0647416
MF	GO:0003774	Cytoskeletal motor activity	9/192	111/18410	2.45e−06	0.0002	− 0.3333333
MF	GO:0035173	Histone kinase activity	4/192	16/18410	1.89e−05	0.0012	1.0000000
MF	GO:0001664	G protein-coupled receptor binding	11/192	288/18410	0.0002	0.0080	− 0.3015113
MF	GO:0036041	Long-chain fatty acid binding	3/192	15/18410	0.0005	0.0152	− 0.5773503

**Table 4:** KEGG analysis

Ontology	ID	Description	Gene ratio	Bg ratio	*P *value	*p*.adjust	*Z* score
KEGG	hsa04110	Cell cycle	11/95	126/8164	1.99e−07	4.18e−05	2.1105794
KEGG	hsa03320	PPAR signaling pathway	7/95	75/8164	2.41e−05	0.0010	− 0.3779645
KEGG	hsa04115	p53 signaling pathway	5/95	73/8164	0.0015	0.0400	1.3416408
KEGG	hsa04062	Chemokine signaling pathway	8/95	192/8164	0.0017	0.0400	0.0000000
KEGG	hsa04060	Cytokine–cytokine receptor interaction	10/95	295/8164	0.0022	0.0457	0.0000000

### Correlation of CD72 expression with tumor immunity

**Fig. 6:**
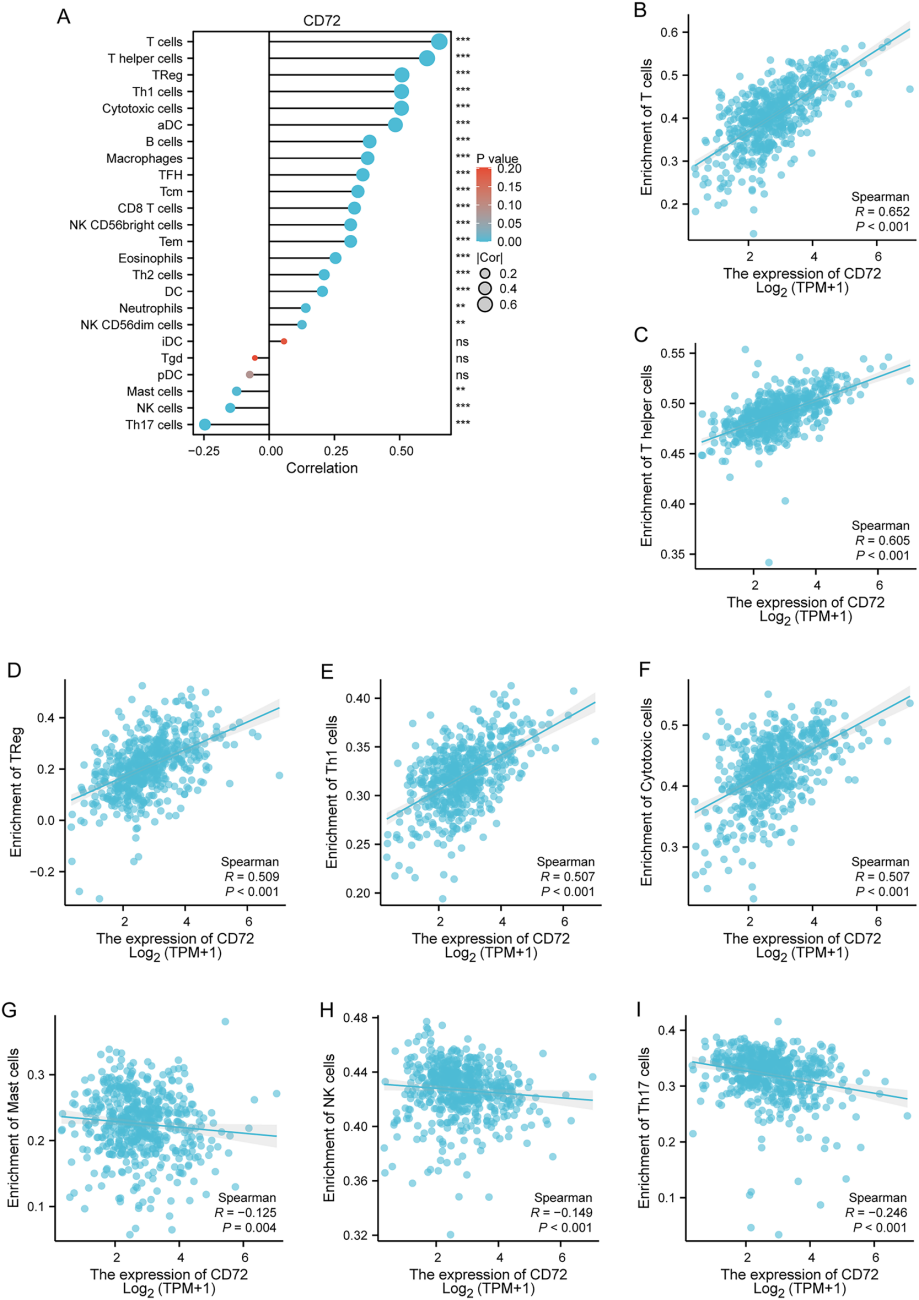
CD72 expression and tumor immunity

**Fig. 7:**
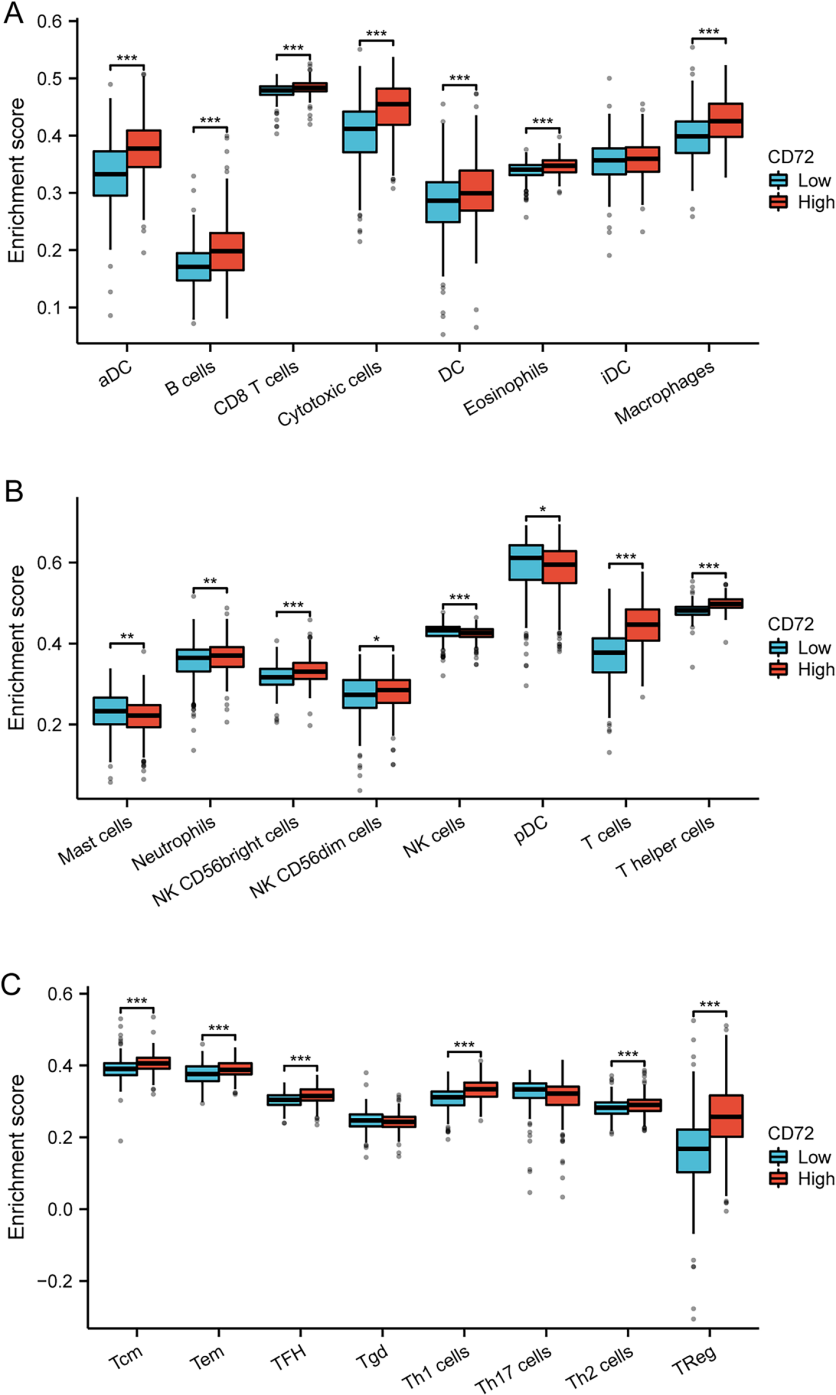
The relationship between CD72 expression and immune cells

**Fig. 8:**
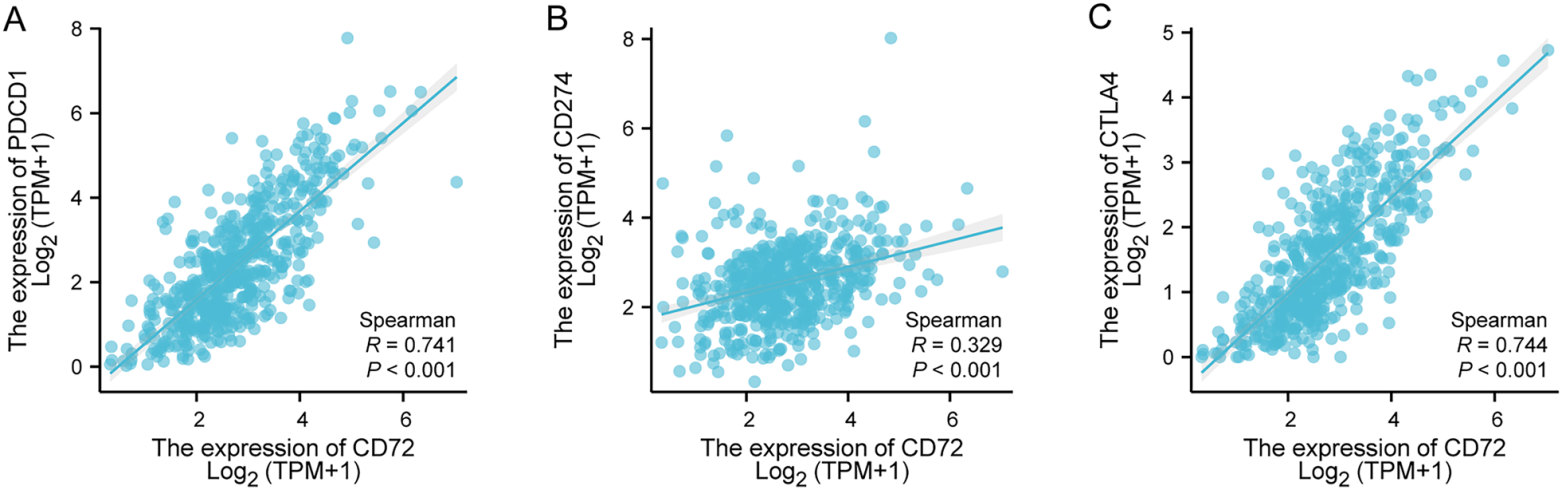
Tumor immune checkpoints and CD72 expression

As shown in Fig. 6A, the relationship between the relative number of 24 immune cells and the expression of CD72 in KIRC was evaluated using the ssGSEA algorithm. As shown in Fig. 6B–*I, different types of immune cells were correlated with CD72 expression, including T cells (*R* = 0.652, *P* < 0.001), T helper cells (= 0.605, *P* < 0.001), TReg (*R* = 0.509, *P* < 0.001), Th1 cells (*R* = 0.507, *P* < 0.001), Cytotoxic cells (*R* = 0.507, *P* < 0.001), Mast cells (*R* = − 0.125, *P* < 0.004), NK cells (*R* = − 0.149, *P* < 0.001), Th17 cells (*R* = 0.246, *P* < 0.001). Mann–Whitney *U* test (Wilcoxon rank sum test) was used to detect the enrichment of immune cells in CD72 high- and low-expression groups. The results showed that compared with the CD72 low-expression group, in the CD72 high-expression group, T cells, T helper cells, Treg, Th1 cells, Cytotoxic cells, aDC, B cells, Macrophages, TFH, Tcm, CD8 T cells, CD56bright cells, Tem, Eosinophils, and CD72 bright cells were enriched. Cells, Tem, Eosinophils, DC, and Neutrophils were more enriched (Fig. 7A–C). In KIRC, the expression of CD72 was positively associated with the expression of PD-1 (PDCD1) (*R* = 0.741, *P* < 0.001), CTLA4 (*R* = 0.744, *P* < 0.001), and PD-L1 (CD274) (*R* = 0.329, *P* < 0.001) using the spearman's analysis (Fig. 8A–C).

### Evaluation of CD72 expression

**Fig. 9:**
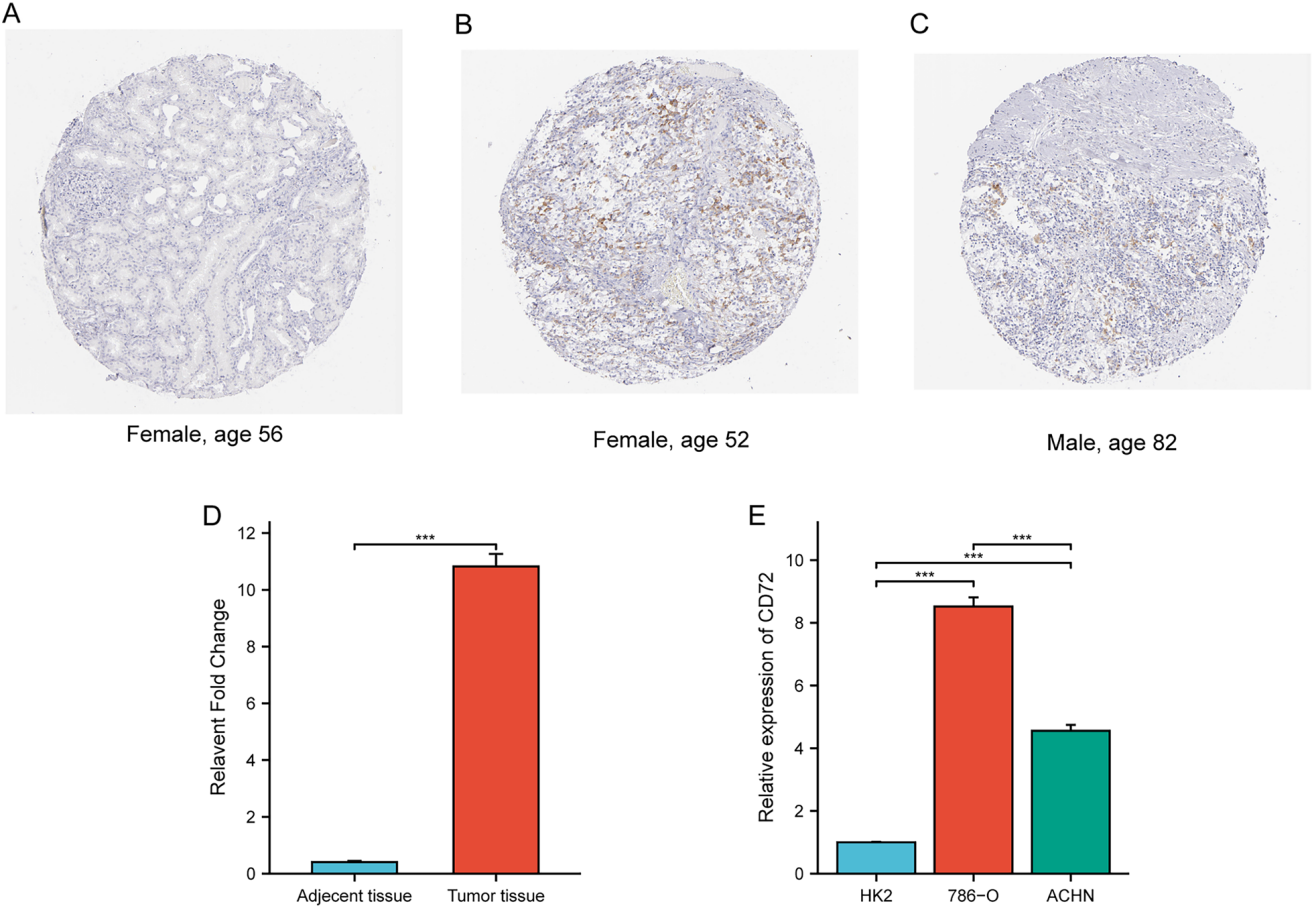
Evaluation of the expression of CD72 in renal clear cell carcinoma cell line

Next, we evaluated the expression of CD72 in KIRC cells 786-O, ACHN, and renal cells HK-2 in Real-time PCR. As shown in Fig. 9A, the expression of CD72 in renal cancer cells was higher than in normal renal cells. In addition, the immunohistochemical results in the HPA database confirmed this result (Fig. 9B, C).

## Discussion

KIRC, as the most common renal cell carcinoma, is challenging to diagnose at an early stage, surgery has limitations, and postoperative metastasis and recurrence are the main reasons for its high mortality rate. With the emergence of tyrosine kinase inhibitors targeting vascular endothelial growth factor receptors, rapamycin-targeted protein inhibitors, and immune checkpoint inhibitors, the choice of second-line treatment is expanding, and renal cancer treatment has entered a new era [43]. However, KIRC has high intrinsic heterogeneity and lacks early diagnostic and prognostic biomarkers [44, 45]. In addition, KIRC is one of the most immune-infiltrated tumors [46, 47]. Therefore, searching for new diagnostic and prognostic biomarkers and therapeutic targets and developing new anti-tumor drugs and immunotherapies are essential ways to improve the survival of KIRC patients.

In this study, we obtained clinical and RNA data of KIRC patients from the TCGA database and downloaded immune-related genes from the ImmPort database. Then we used the "limma" package, "survival" package, and Venn overlaps analysis to obtain differentially expressed mRNAs (DEmRNAs) related to the prognosis and immunity of KIRC patients, and we finally chose CD72 as the target gene.

Our study was analyzed using the UCSC XENA and TCGA database, and the results showed that in KIRC, compared with paracancerous tissues, the expression level of CD72 was significantly increased, which correlated with poor patient prognosis. KIRC patients with high expression of CD72 were less likely than those with low expression of CD72 in terms of either OS, DSS, or PFI. These results suggest that CD72 is associated with the occurrence and development of KIRC. In this study, CD72 expression was significantly correlated with the Pathologic T stage, Pathologic stage, Pathologic M stage, Pathologic N stage, Histologic grade, Laterality, and OS event, suggesting that CD72 may play an essential role in the biological function of tumor cells. Play an important role in the biological function of tumor cells. These results suggest that CD72 may be a useful diagnostic molecular marker for KIRC and can predict the outcome of KIRC patients. In addition, based on the ROC diagnostic curve (AUC = 0.954) and logistic analysis, it was further shown that CD72 could be used for KIRC diagnosis.CD72 is expected to be a potential biomarker for determining the prognosis, suggesting that patients with KIRC may benefit from using CD72 for diagnosis and prognosis.

To further understand the molecular mechanism of CD72 in tumorigenesis and development, functional enrichment analysis of GO, KEGG, and GSEA was performed using CD72 and its related differentially expressed genes. GO-based enrichment analysis showed that CD72 and its co-expressed mRNAs were enriched for several molecular functions (e.g., microtubule binding, cytoskeletal motor activity, histone kinase activity, G protein-coupled receptor binding, long-chain fatty acid binding), biological processes (mitotic nuclear division, mitotic sister chromatid segregation, organelle fission, chromosome segregation).

Abnormal expression of microtubule-binding proteins can cause cytoskeletal changes. Abnormal cytoskeletal motility activity may disrupt critical processes such as cell proliferation, migration, and metastasis [48, 49], leading to disorganization of cellular structure and abnormal proliferation of tumor cells [50]. Aberrant activation of histone kinases may lead to disrupted chromatin structure and aberrant gene expression, thereby affecting tumor cell proliferation and transcriptional regulation [51, 52]. Aberrant activity of G protein-coupled receptors may trigger aberrant activation of cell signaling pathways, promoting the proliferation and survival of tumor cells [53, 54]. RCC is essentially a metabolic disease characterized by a reprogramming of energetic metabolism [55–58]. In particular the metabolic flux through glycolysis is partitioned [59–61], and mitochondrial bioenergetics and OxPhox are impaired, as well as lipid metabolism [59, 62–64]. In addition, aberrant expression of long-chain fatty acid binding proteins may lead to disturbances in intracellular energy metabolism and alterations in tumor cell growth [65].

In tumor development, mitotic nuclear division, sister chromatid segregation, and abnormal chromosome segregation lead to genomic instability, increase the risk of chromosomal abnormalities and mutations, and promote tumor formation and development [66, 67]. The correct alignment of the mitotic spindle during cell division is critical for cell fate determination, tissue organization, and development. Changes in the dynamics and control of microtubules that impair the mitotic spindle lead to chromosomal instability, leading to the generation of tumor cells [68, 69]. The molecular mechanism of CD72 in the development of KIRC may be related to its role in cell cycle regulation, cell growth, and cell migration.

KEGG analysis also showed that CD72 function is associated with signaling pathways such as cell cycle, PPAR signaling pathway, p53 signaling pathway, coagulation factor signaling pathway, and cytokine–cytokine receptor interactions. Abnormal cell cycle regulation is essential in tumorigenesis and progression, leading to unlimited cell proliferation [70]. Abnormalities in PPAR and p53 signaling pathways are associated with the progression of multiple tumors [71–75]. Abnormalities in chemokine pathways are associated with tumor infiltration, metastasis, and neovascularization [76–78]. Cytokine-receptor interactions are critical for regulating cell growth, differentiation, and immunity, and abnormalities can lead to tumor immune escape phenomena [79, 80].

Based on GSEA enrichment analysis, CD72 and its co-expressed mRNAs are enriched in signaling pathways such as Pd 1 Signaling, CTLA4 Pathway, Th17 Cell Differentiation Pathway, B Cell Receptor Signaling Pathway, Costimulation By the CD28 Family, Primary Immunodeficiency, Cancer Immunotherapy By Pd1 Blockade, Modulators of Tcr Signaling and T Cell Activation, Costimulation By the CD28 Family, Primary Immunodeficiency, Cancer Immunotherapy By Pd1 Blockade, Modulators of Tcr Signaling and T Cell Activation, and other Signaling Pathways associated with tumor immunity and tumorigenesis.

Correlation analysis showed a significant association between CD72 and PD-L1 (CD274) and CTLA4. Pd 1 and CTLA4 are two key immune checkpoint molecules with essential roles in regulating T-cell function and activating [81, 82]. When Pd-L1 (ligand for Pd 1) and CTLA4 are upregulated, tumor cells can evade the immune response and promote tumor growth. Activated T cells express PD-1 encoded by the PDCD1 gene, while PD-L1 encoded by the CD274 gene is overexpressed on the membrane surface of tumor cells. PD-1 binds to PD-L1, inhibits T-cell activation and causes their death, and then assists in the immune escape of tumor cells [83]. Abnormal B-cell receptor (BCR) signaling pathway activation may also lead to malignant transformation of B cells, which drives tumorigenesis and progression [84]. In addition, through activation of the Th17 cell differentiation pathway, increased numbers of tumor-infiltrating immune cells, promotion of neovascularization, and alterations in the tumor microenvironment can promote tumorigenesis and progression [85]. Co-stimulatory pathways of the CD28 family impact tumor growth and immune surveillance by influencing T-cell activation, proliferation, and anti-tumor immune responses, as well as modulating immune cell function [86]. The above results suggest that CD72 is essential in tumorigenesis and progression.

The results of immune infiltration showed that the degree of infiltration of T cells, T helper cells, Treg, Th1 cells, cytotoxic cells, aDC, B cells, macrophages, TFH, Tcm, CD8 T cells, CD56bright cells, Tem, eosinophils, DC, and neutrophils showed a significant positive correlation with CD72 expression. Mast cells, NK cells, and Th17 infiltration degree were significantly negatively correlated with CD72 expression. The transition from Th1/Th2 balance to Th2 dominance is critical during tumor progression. Th2 cells are detrimental to cellular immune anti-tumor effects. Restoration of Th1/Th2 balance is vital in tumor therapy [87]. Tregs are usually enriched in the tumor microenvironment, and many Tregs have a poor prognosis [88]. Lack of NK cell numbers and defective NK cell function promote the escape of tumor cells from immune surveillance [89]. Tumor-Associated Macrophages (TAMs) Promote Tumor Growth and Metastasis by Suppressing Tumor Immunosurveillance [90]. In addition, immunomodulatory interactions may be altered between lymphocytes and non-lymphocytes, leading to immune escape, immunosuppression, tumor growth and progression promotion, and concomitant suppression of tumor immunotherapy in KIRC [90]. All these results suggest that up-regulation of CD72 expression can suppress the anti-tumor immune response in KIRC patients.

In this study, correlation analysis showed a significant association between CD72 and PD-L1 (CD274), CTLA4, PD-1 (PDCD1), and immune cells (e.g., T cells, T helper cells, TReg, Th1 cells, Cytotoxic cells, Mast cells, NK cells, Th17 cells, etc.) were significantly associated with each other. In addition, in KIRC patients, compared with the CD72 low-expression group, the CD72 high-expression group showed a significant correlation between immune cells (T cells, T helper cells, Treg, Th1 cells, Cytotoxic cells, aDC, B cells, Macrophages, TFH, Tcm, CD8 T cells, CD56bright cells, Tem, Eosinophils, DC, Neutrophils, etc.) were more enriched. Our results suggest that CD72 is closely associated with immune infiltration and immunosuppression in the microenvironment of KIRC tumors.

## Conclusion

In conclusion, we found that CD72 was overexpressed in KIRC and correlated with the clinical stage of patients, and it may be a marker for early diagnosis of KIRC patients. In addition, CD72 was associated with poor patient prognosis and could be an independent prognostic factor for KIRC.CD72 may promote tumor development by regulating the cell cycle and immune-related signaling pathways and facilitating immune cell infiltration.CD72 also showed a significant positive correlation with PD-L1 (CD274), CTLA4, and PD-1 (PDCD1) immunotherapeutic targets. Therefore, CD72 is expected to be a potential diagnostic and prognostic biomarker for KIRC and a new target for anti-tumor drug development. However, the results obtained in this study require additional experiments, such as animal and cellular experiments, to further validate the mechanism by which CD72 promotes KIRC. Since this study was a retrospective study based on the available RNA sequencing data in the TCGA and GEO databases, prospective studies are needed in the future to minimize the bias caused by retrospective studies.

## Data Availability

The datasets generated and/or analyzed during the current study are available in the GEO (https://www.ncbi.nlm.nih.gov/geo/), ImmPort (https://www.immport.org/shared/ home), UCSC XENA (https://xenabrowser.net/datapages/), TCGA (https://portal.gdc.cancer.gov), and HPA (https://www.proteinatlas.org/) repositories.

## Abbreviations

KIRC: Kidney clear cell carcinoma
TCGA: The Cancer Genome Atlas
GEO: Gene Expression Omnibus
GO: Gene Ontology
KEGG: Kyoto Encyclopedia of Genes and Genomes
GSEA: Gene set enrichment analysis
DEGs: Differentially expressed genes
OS: Overall survival
ANOVA: One-factor analysis of variance
IHC: Immunohistochemical
